# Retrospective assessment of short-term outcomes of robotic- versus laparoscopic-assisted duodenal diamond anastomosis in neonates

**DOI:** 10.1007/s00464-024-11070-9

**Published:** 2024-07-24

**Authors:** Si-Si Yang, Chengjie Lv, Shoujiang Huang, Jin-Fa Tou, Deng-Ming Lai

**Affiliations:** Department of Neonatal Surgery, National Clinical Research Center for Child Health, Children’s Hospital, Zhejiang University School of Medicine, 3333 Binsheng Rd, Hangzhou, Zhejiang China

**Keywords:** Pediatrics, Robotic surgery, Laparoscopy, Neonatal duodenal obstruction

## Abstract

**Objective:**

The purpose of this study was to retrospectively compare the short-term outcomes of robotic- (RAD) and laparoscopic-assisted duodenal diamond-shaped anastomosis (LAD) in neonates.

**Methods:**

Neonates who underwent RAD (*n* = 30) or LAD (*n* = 38) between January 2019 and December 2022 were analyzed retrospectively. Major patient data were collected, including preoperative, intraoperative, and postoperative information.

**Results:**

All patients were neonates below the age of 30 days weighing 4 kg. Thirty (44.1%) neonates underwent RAD and 38 neonates (55.9%) underwent LAD. Compared to the LAD group, the RAD group had a shorter intra-abdominal operation time (RAD, 60.0(50.0 ~ 70.0) min; LAD, 79.9(69.0 ~ 95.3) min; *p* < 0.001). There were no significant differences in immediate and 30-day complications between the two groups.

**Conclusions:**

RAD is safe and effective in neonates. Compared to traditional LAD, RAD showed comparable results.

**Graphical abstract:**

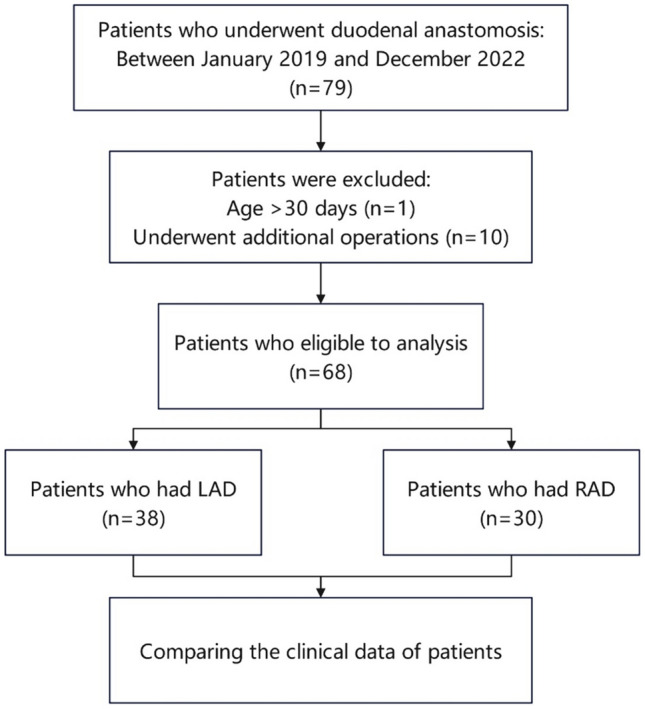

Neonatal duodenal obstruction is a congenital abnormality that can be diagnosed and characterized by postnatal vomiting. Since the surgical technique was published in 2001–2002 [1], laparoscopic-assisted duodenal diamond-shaped anastomosis (LAD) has become popularized for the treatment of neonatal duodenal obstruction. Its safety and efficacy have been proven by various retrospective case series [2–4]. However, the outcomes and the rate of conversion to open surgery would vary with the surgeon’s surgical experience because the limited abdominal cavity volume of neonates is technically demanding and requires a steep learning curve. Robotic surgery has the advantages of three-dimensional high-definition visualization, stable and dexterous control, and faster learning curve. This new technology has gained popularity in adult surgery; however, robotic surgery for children has been less widely reported, especially in infants of low weight and young age. Arellano et al. reported a series of pediatric surgeries in young infants, aged 3.5–204 months and weighing 5–102 kg [5]. The technical limitations of robotic surgery were more pronounced when the patient weighed less than 3 kg [6, 7].

Our department has completed 30 cases of robotic-assisted diamond-shaped anastomosis (RAD) using the da Vinci Xi Surgical System and 38 cases of LAD from January 2019 to December 2022. All patients were neonates below the age of 30 days weighing 4 kg. In this retrospective analysis, we present our experience with RAD in neonates and analyze the short-term outcomes of patients following RAD and LAD.

## Methods

### Study population

The medical records of 30 neonates who underwent RAD and 38 neonates who underwent LAD between January 2019 and December 2022 were enrolled in this retrospective study, which was approved by the Institutional Review Board of the Children’s Hospital of Zhejiang University School of Medicine (approval number: 2023-IRB-0257-P-01).

The inclusion criteria were as follows: (1) patients with a definite diagnosis of neonatal duodenal obstruction, (2) patients who underwent surgery for duodenal diamond-shaped anastomosis at our center, and (3) the parents of the pediatric patient signed the informed consent for the operation.

The exclusion criteria were as follows: (1) patients who required additional operations other than duodenal anastomosis within the same hospital stay and (2) patients who were > 30 days.

After providing detailed information on LAD and RAD for neonatal duodenal obstruction, families are permitted to make the final decision on which surgical approach to use for their neonates.

### Data collection

Major data of the patients with neonatal duodenal obstruction were collected, including preoperative, intraoperative, and postoperative information.

Preoperative information included neonatal characteristics, such as sex, gestational age, age, weight, height, and body mass index (BMI). Notably, age, weight, height, and BMI were all obtained during the operation.

Intraoperative information included the total operation time, anesthesia time, fluid input, and urine output. The docking time, intra-abdominal operation time (console time), anastomosis time of the RAD, intra-abdominal operation time, and anastomosis time of the LAD were recorded.

The postoperative information included the duration of NICU stay, mechanical ventilation, antibiotic therapy, time to taking water, time to starting milk, immediate and 30 day complications, length of hospital stay, and hospitalization expenses. Immediate complications [8] were defined as those that occurred during hospitalization, including anastomotic leakage, emesis, and infection. 30 day complications were defined as complications within 30 days of discharge, including anastomotic leakage, emesis, and incision infection.

### Surgical procedures

#### LAD

**Fig. 1:**
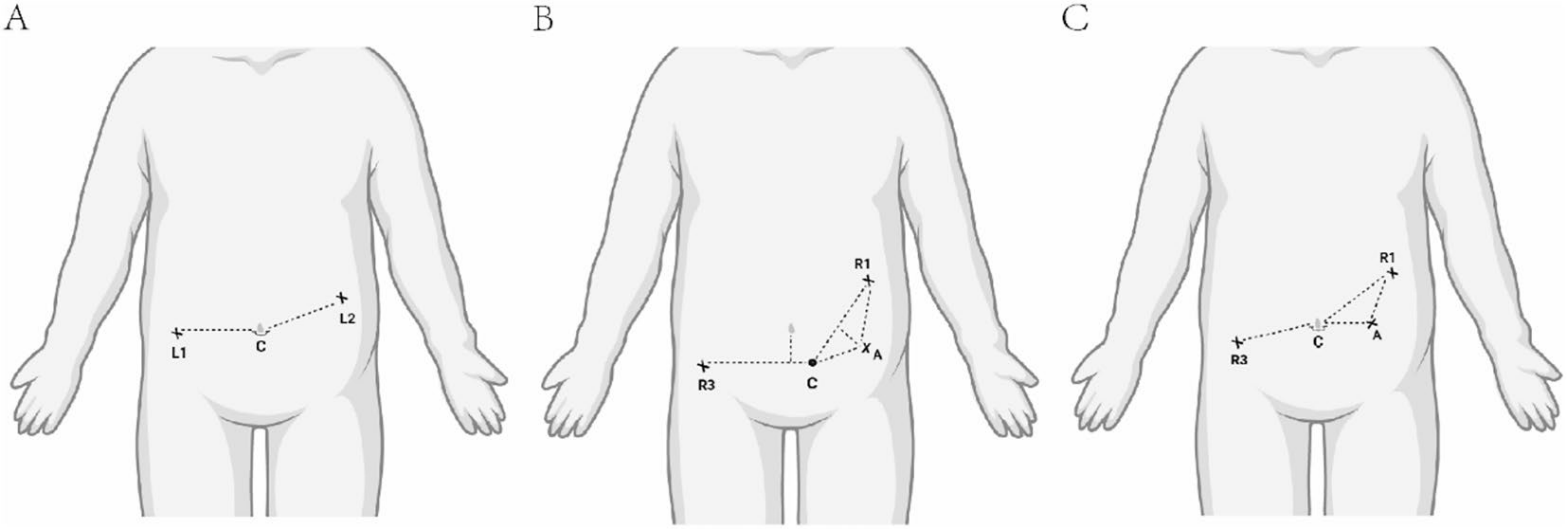
Port placement in diamond-shaped anastomosis. **A** Laparoscopic-assisted duodenal diamond-shaped anastomosis. **B** Robotic-assisted diamond-shaped anastomosis for neonates (BW < 3 kg). **C** Robotic-assisted diamond-shaped anastomosis for neonates (BW ≥ 3 kg). L laparoscopic port, R robotic arm, C camera, A assistant

LAD for neonatal duodenal obstruction is considered to be the gold standard surgical method used in many centers [9, 10]. The patients were positioned under general anesthesia on an operating table. The surgeon stood at the tail side of the patient and the monitor was placed on the head side of the patient. Three trocars were inserted, a 5 mm port was placed at the umbilicus for a 30° camera and two 3 mm ports were placed on either side of the umbilicus (Fig. 1a). The proximal duodenum was opened transversely and the distal duodenum was opened longitudinally. A diamond-shaped anastomosis is generally accomplished using the technique introduced by Kimura et al. [11].

#### RAD

After the induction of general anesthesia, the patient remained in a supine position with the head high and feet low tilted approximately 30°, head, neck, and trunk height 5–10 cm. A trocar was set as the endoscopic port (No. 2 arm), located in the left lower abdomen at the intersection of the transverse stripes of the abdomen and the midline of the clavicle (BW < 3 kg) (Fig. 1b) or at the umbilicus (BW ≥ 3 kg) (Fig. 1c), with a 5 mmHg pneumoperitoneum pressure. Using camera images, a trocar was placed on the left upper abdomen, near the left anterior axillary line, 1 cm below the ribs, and another trocar was placed on the right lower abdomen, where the abdominal transverse stripes met the anterior axillary line. Arms 1 and 3 were then introduced, and a trocar was placed at the lower left of the midpoint of the connection between the endoscopic port and No. 1 arm, as far away as possible from the operation area. The procedures were the same as those for the LAD. Finally, a 5–0 double-needle absorbable suture was used to suture the walls of the two incisions continuously.

### Statistical analysis

Categorical variables are presented as frequency and percentage. Fisher’s exact test was used to compare categorical data. Continuous variables were analyzed using the Shapiro–Wilk test and presented as means, standard deviations, or medians and interquartile ranges. The Mann–Whitney *U* test was used to analyze continuous data, such as age, height, operation time, fluid input, urine output, blood loss, duration of NICU and antibiotic therapy, time to having water and milk, length of hospital stay, and hospitalization expenses. The *T*-test was used to analyze continuous data, such as gestational age, weight, BMI, anesthesia time, fluid input rate, and duration of mechanical ventilation. Statistical significance was defined as a two-sided *p* < 0.05. Statistical analyses were performed using the SPSS software version 26.0.

## Results

There were 79 patients with neonatal duodenal obstruction were diagnosed and treated at our institution during the study period between January 2019 and December 2022. Eleven patients were excluded from the analysis based on the exclusion criteria. The remaining 68 patients had available medical records that could be used for the analysis. None of the patients had severe congenital malformation. Overall, 30(44.1%) and 38(55.9%) neonates underwent RAD and LAD procedures, respectively. All patients were discharged from the hospital and were followed up for 30 days.

The demographic characteristics of patients are shown in Table 1. There were no significant differences between the LAD and RAD groups in terms of sex, gestational age, weight, height, and BMI. The age at the time of surgery ranged from 1 to 26 days, all of which were in the neonatal period (≤ 28 days). Weight at the time of surgery ranged from 1.3 to 4.0 kg, with a median weight of 2.7 kg. 23(33.8%) preterm infants underwent surgery: 10 in the RAD group and 13 in the LAD group. Among the youngest patients at 1-day-old, three underwent RAD and two underwent LAD. The smallest number of patients in the LAD group was 2, weighing 1.3 kg, and in the RAD group it was 1, weighing 1.6 kg.

**Table 1:** Patient characteristics according to surgical approach

Group	LAD (*n* = 38)	RAD (*n* = 30)	*p* Value
Gender, *n*(%)			0.468
Female	19	18	
Male	19	12	
Gestational age, weeks	37.4 ± 2.3	37.7 ± 1.5	0.500
Premature, *n*(%)	13(34.2%)	10(33.3%)	1.000
Age, days	4.0(3.0 ~ 6.3)	4.0(2.0 ~ 7.3)	0.955
Weight, kg	2.7 ± 0.7	2.7 ± 0.6	0.956
Height, m	0.5(0.5 ~ 0.5)	0.5(0.5 ~ 0.5)	0.864
BMI, kg/m2	11.0 ± 1.9	11.2 ± 1.9	0.673

*BMI* body mass index

The intraoperative outcomes are presented in Table 2. The mean total operating time in the RAD group and the LAD group were 100.0(90.0 ~ 120.0) and 92.5(80.0 ~ 110.0) minutes, respectively. The operative time was significantly longer in the RAD group than in the LAD group (*p* = 0.020). However, the intra-abdominal operation time was significantly shorter in the RAD group than in the LAD group (*p* < 0.001). Anastomosis and docking times accounted for 25% and 15% of the total surgical time in the RAD group, respectively. In contrast, the anastomosis time was 12.5% in the LAD group and the time to establish pneumoperitoneum was negligible. However, there were no significant differences between the LAD and RAD groups with respect to the anesthesia time. In addition, fluid input rates were similar between the two groups, while urine output rates were lower in the RAD group than in the LAD group; however, the difference was not significant.

**Table 2:** Intraoperative outcomes according to surgical approach

Group	LAD (*n* = 38)	RAD (*n* = 30)	*p* Value
Total operation time (min)	92.5(80.0 ~ 110.0)	100.0(90.0 ~ 120.0)	0.020
Docking time (min)		15.0(12.0 ~ 18.5)	–
Intra-abdominal operation time (min)	79.9(69.0 ~ 95.3)	60.0(50.0 ~ 70.0)	< 0.001
Anastomosis time (min)	11.6(10 ~ 13.8)	25.0(22.0 ~ 30.0)	< 0.001
Anesthesia time (min)	155.3 ± 24.5	157.5 ± 18.6	0.680
Total fluid input (mL)	87.5(58.75 ~ 100)	67.5(50 ~ 100)	0.357
Fluid input rate (mL/kg/hr)	11.9 ± 3.7	11.0 ± 4.0	0.348
Total Urine output (mL)	10.0(5.0 ~ 20.0)	10.0(5.0 ~ 10.0)	0.151
Urine output rate (mL/kg/hr)	1.5(0.9 ~ 2.2)	1.2(0.9 ~ 1.5)	0.079

Postoperative data, hospitalization costs, and complications are summarized in Table 3. The length of the NICU stay was similar between the two groups. The duration of mechanical ventilation, antibiotic therapy, hospital stay, time to drinking water, and time to starting milk were all longer in the RAD group than in the LAD group; however, the differences were not statistically significant. The costs of the surgical procedure were significantly higher in the RAD group than in the LAD group (*p* < 0.001), but there were no significant differences in the other hospitalization expenses apart from the operation. In the RAD group, 8 patients developed complications during hospitalization, including 1 (minor anastomotic leakage, emesis, and infection), 4 (emesis), and 3 (infection). Seven patients developed complications during the hospital stay in the LAD group: 1 (minor anastomotic leakage), 4 (emesis), 1 (infection), and 1(emesis and infection). In this study, four patients in the RAD group and two patients in the LAD group had occasional vomiting within 30 days of follow-up without anastomotic leakage or incision infection. In summary, the number of immediate and 30-day complications in the RAD group was higher than that in the LAD group; however, the difference was not significant.

**Table 3:** Postoperative outcomes and complications according to surgical approach

Group	LAD (*n* = 38)	RAD (*n* = 30)	*p* Value
NICU length of stay (days)	0.9(0.8 ~ 2.7)	0.9(0.7 ~ 1.6)	0.396
Mechanical ventilation (hours)	10.9 ± 5.3	12.1 ± 5.9	0.385
Antibiotic therapy (days)	8.5(6.0 ~ 10.0)	10.0(7.0 ~ 11.5)	0.089
Time to taking water (days)	5.5(4.0 ~ 8.0)	7.0(4.8 ~ 7.3)	0.270
Time to starting milk (days)	6.0(4.0 ~ 7.0)	7.0(5.0 ~ 7.3)	0.228
Post-operative hospital stay (days)	12.0(10.0 ~ 18.0)	15.5(11.8 ~ 21.0)	0.213
Total hospital stay (days)	16.5(13.8 ~ 23.0)	20.0(15.8 ~ 25.5)	0.218
Total hospitalization expenses (thousand yuan)	31.9(24.7 ~ 39.8)	78.8(70.3 ~ 84.1)	< 0.001
Total expenses of surgical procedure (thousand yuan)	4.6(3.7 ~ 5.8)	47.6(46.6 ~ 47.6)	< 0.001
Hospitalization expenses apart from operation (thousand yuan)	26.5(19.4 ~ 34.0)	32.2(23.2 ~ 37.7)	0.142
Immediate complications, *n* (%)	7(18.4%)	8(26.7%)	0.557
Stomal leakage	1(2.6%)	1(3.3%)	1.000
Emesis	5(13.2%)	5(16.7%)	0.740
Infection	2(5.3%)	4(13.3%)	0.394
30 day complications, *n* (%)	2(5.3%)	4(13.3%)	0.394
Stomal leakage	0	0	–
Emesis	2(5.3%)	4(13.3%)	0.394
Incision infection	0	0	–

## Discussion

In recent years, robot-assisted surgery in children has achieved great success, including radical choledochal cyst resection [12] and ureteral replantation [13, 14]. The advantages of robotic surgery for pediatric surgical diseases have become increasingly prominent. However, the application of robotic surgery in pediatric general surgery is in its nascent stage, particularly in neonatal surgery. This study is the first to compare LAD and RAD to demonstrate the effectiveness and safety of RAD in neonatal patients. In terms of surgical outcomes and short-term prognosis, EAD is safe in neonates. This paper aims to summarize and share the valuable experience of robotic surgery in the treatment of duodenal obstruction in newborns aged < 30 days.

The establishment of a surgical medical team is essential for the safe use of the Da Vinci system, including surgical and nursing teams. The Da Vinci surgical team needed to undergo intuitive surgical robotic training, divided into two stages of pre-clinical and clinical training, and finally obtained the qualification certificate of da Vinci surgery robotic surgery issued by Intuitive Surgical, Inc., authorized by the National Health Planning Commission. Nurses with rich experience in laparoscopic surgery were selected to participate in the professional training of the Da Vinci robotic surgery system and obtain professional certificates after passing the assessment. In addition, entry and exit of personnel were strictly controlled. Except for the surgical medical team, the other personnel were not allowed to visit the robot operating room. To the surgical medical team, the docking time was controlled at 12.0 ~ 18.5 min, and the anastomosis time was controlled at 22–30 min. However, owing to the additional robot installation and disassembly time, the operation time was significantly increased compared to laparoscopic surgery (RAD, 100.0(90.0 ~ 120.0), LAD, 92.5(80.0 ~ 110.0), *p* = 0.020).

Duodenal diamond-shaped anastomosis was elected as our early robotic case based on the following points: (1) the duodenum is relatively fixed and located on the right side of the upper abdomen, which increases the operating space and provides favorable conditions for neonatal robotic surgery; (2) the surgeon at the console was good at doing these cases by the standard laparoscopic approach; (3) a simple case was more beneficial to the entire robotic team, as everyone became adept at mastering the robot; and (4) simple operation, 3D surgical field, is an ideal introductory teaching case for residents.

In this study, the age and weight of patients in the RAD group were similar to those in the LAD group. We did not restrict the application of RAD in very young or underweight patients, although this was not recommended in some reports [6, 8]. Indeed, the limited abdominal operation space of neonates is the greatest limitation of RAD. When placing the trocars in the RAD, the largest distance is chosen to avoid mutual interference between the instrument arms and the central camera. The holes were positioned more laterally than the typical location for the LAD, especially in children weighing less than 3000 g. Interestingly, the intra-abdominal operation time was significantly shorter in the RAD group than in the LAD group (RAD, 60.0(50.0 ~ 70.0)min; LAD, 79.9(69.0 ~ 95.3)min; *p* < 0.001). We believe that improvements in technology and further miniaturization of robotic arms will make RAD even more advantageous. The essence of RAD is laparoscopic surgery with upgraded instruments and equipment; its operating principles and procedures are the same. The robot’s 3D surgical field is clearer, and the instrument arms are fixed, which can prevent the lens from being blocked and ensure a smooth operation. The da Vinci robotic arm system is highly flexible and can complete difficult operations, such as grasping, holding, suturing, and ligation, in a limited space [15]. In addition, the robotic arms can filter out slight shaking of the hand, which is more conducive to the accurate positioning of the incision and suture site. Compared with the traditional LAD, the surgeon’s fatigue was reduced, satisfaction was improved, adaptation was faster, and the learning curve was significantly lower. However, the disadvantages of robotic surgery are obvious. The trocar position selection was more limited than LAD, and the operation cost was higher than LAD (RAD, 47.6(46.6 ~ 47.6), LAD, 4.6(3.7 ~ 5.8), *p* < 0.001), which will increase medical costs. Owing to the high cost of robotic surgery, its use in simple procedures, such as RAD, is still questionable.

There were three surgical options for duodenal obstruction, including open surgery, laparoscopic surgery and robotic surgery. Compared with open surgical repair, laparoscopy was more popular with parents because of its cosmetic benefits [9]. In addition, the duration of total enteral feeding and postoperative hospital stay were reduced in laparoscopic repair [16]. The outcomes and complications in the LAD group and RAD group were equivalent.

Although the feasibility, safety, and effectiveness of RAD were demonstrated in our study, we do not recommend RAD in patients weighing less than 2000 g based on experience. In addition, the application of robotic surgery in neonates requires further prospective studies. We believe that, as robotic surgery systems are further developed, more studies in the future will support the development of robotic surgery in neonates and yield more positive outcomes.

## Conclusion

The da Vinci surgical system is safe and feasible for the treatment of duodenal obstruction in neonates below the age of 30 days.
